# Cycle Inhibiting Factors (CIFs) Are a Growing Family of Functional Cyclomodulins Present in Invertebrate and Mammal Bacterial Pathogens

**DOI:** 10.1371/journal.pone.0004855

**Published:** 2009-03-24

**Authors:** Grégory Jubelin, Carolina Varela Chavez, Frédéric Taieb, Mark J. Banfield, Ascel Samba-Louaka, Rika Nobe, Jean-Philippe Nougayrède, Robert Zumbihl, Alain Givaudan, Jean-Michel Escoubas, Eric Oswald

**Affiliations:** 1 INRA, UMR1225, Toulouse, France; 2 Université de Toulouse, ENVT, UMR 1225, Toulouse, France; 3 INRA, UMR 1133 Laboratoire EMIP, Montpellier, France; 4 Université Montpellier 2, UMR 1133 Laboratoire EMIP, Montpellier, France; 5 Department of Biological Chemistry, John Innes Centre, NR4 7UH, Norwich, United Kingdom; University of Liverpool, United Kingdom

## Abstract

The cycle inhibiting factor (Cif) produced by enteropathogenic and enterohemorrhagic *Escherichia coli* was the first cyclomodulin to be identified that is injected into host cells *via* the type III secretion machinery. Cif provokes cytopathic effects characterized by G_1_ and G_2_ cell cycle arrests, accumulation of the cyclin-dependent kinase inhibitors (CKIs) p21^waf1/cip1^ and p27^kip1^ and formation of actin stress fibres. The X-ray crystal structure of Cif revealed it to be a divergent member of a superfamily of enzymes including cysteine proteases and acetyltransferases that share a conserved catalytic triad. Here we report the discovery and characterization of four Cif homologs encoded by different pathogenic or symbiotic bacteria isolated from vertebrates or invertebrates. Cif homologs from the enterobacteria *Yersinia pseudotuberculosis*, *Photorhabdus luminescens*, *Photorhabdus asymbiotica* and the β-proteobacterium *Burkholderia pseudomallei* all induce cytopathic effects identical to those observed with Cif from pathogenic *E. coli*. Although these Cif homologs are remarkably divergent in primary sequence, the catalytic triad is strictly conserved and was shown to be crucial for cell cycle arrest, cytoskeleton reorganization and CKIs accumulation. These results reveal that Cif proteins form a growing family of cyclomodulins in bacteria that interact with very distinct hosts including insects, nematodes and humans.

## Introduction

Pathogenic bacteria have developed sophisticated arsenals of virulence factors that hijack eukaryotic host functions to their own benefit. One of the pathways targeted by several bacterial effectors is the eukaryotic cell cycle. These toxins, termed cyclomodulins, can promote cell proliferation or, conversely, inhibit cell growth and modulate differentiation by blocking cell cycle progression [1], [2]. The Cycle Inhibiting Factor (Cif) is a cyclomodulin injected into eukaryotic cells by the type III secretion system (T3SS) of enteropathogenic and enterohemorrhagic *Escherichia coli* (EPEC and EHEC). Cif from pathogenic *E. coli* triggers an irreversible cytopathic effect characterized by cell cycle arrests at the G_2_/M and G_1_/S phase transitions and, at least in HeLa cells, reorganization of the actin network [3]–[6]. In contrast to other cyclomodulins such as the cytolethal distending toxin [7] or colibactin [8], Cif is not a genotoxin nor an activator of DNA-damage checkpoint pathways that lead to phosphorylation of cyclin-dependent kinase 1 and consequent G_2_-arrest [5]. Both G_1_ and G_2_ arrests induced by Cif are correlated with the accumulation of the cyclin-dependent kinase inhibitors (CKIs) p21^waf1/cip1^ and p27^kip1^ (hereafter referred as p21 and p27), which actively participate in the control of cell cycle progression. These accumulations result from inhibition of their proteasome-mediated degradation [6].

Cif is composed of a C-terminal active domain (residues 21–282) and an exchangeable N-terminal translocation signal encoded by the first ∼20 amino acids [9]. The crystal structure of a truncated form of EPEC Cif (lacking the first 99 amino acids) was recently determined. The presence of a conserved catalytic triad comprising Cys109, His165 and Gln185, revealed that Cif is a divergent member of a superfamily of enzymes that includes cysteine proteases, acetyltranferases and transglutaminases [10]. The three amino acids that comprise the triad are essential for Cif's ability to induce cytopathic effects in eukaryotic cells as mutation of these residues leads to loss of function [10].

In EPEC and EHEC, Cif is not encoded within the locus of enterocyte effacement (LEE), which includes T3SS machinery genes and other effectors, but by a temperate lambdoid phage [11]. The *cif* gene has been widely disseminated by phage conversion within the natural population of *E. coli*, but positively selected within LEE-encoding strains [11]. Since Cif targets the cell cycle, a fundamental process conserved in all eukaryotic cells, it is reasonable to speculate that Cif homologs contribute to the pathogenicity of other bacterial species.

In the present study, four homologs of Cif have been identified and characterized in pathogenic or symbiotic bacteria: *Burkholderia pseudomallei*, *Yersinia pseudotuberculosis*, *Photorhabdus luminescens* and *Photorhabdus asymbiotica*. The four Cif homologs are functional and induce cell cycle arrest, p21 and p27 accumulation and actin cytoskeleton rearrangement in HeLa cells in an identical manner to EPEC Cif. The catalytic triad identified in the EPEC Cif crystal structure is strictly conserved in the homologs (at the sequence level) and is involved in their cytopathic activity since mutation of the critical cysteine residue leads to loss of function. Therefore, Cif proteins form a conserved family of cyclomodulins present in both symbionts and pathogens of vertebrate and invertebrate hosts.

## Results

### Genes encoding Cif-like proteins are present in the genomes of *Yersinia*, *Burkholderia* and *Photorhabdus* species

The cyclomodulin Cif was initially identified and characterized in pathogenic *E. coli* (Cif_Ec_) [3]. Sequence database searches using BLAST [12] revealed that Cif_Ec_ shares similarity with hypothetical proteins encoded by the genome of four other bacterial species (Table 1). Cif_Ec_ exhibited a high degree of similarity with Ypk1971 (56% identity), a protein encoded by the human pathogen *Yersinia pseudotuberculosis* strain YPIII [13]. *Y. pseudotuberculosis* infection in humans causes gastroenteritis characterized by a self-limited mesenteric lymphadenitis that mimics appendicitis. Cif_Ec_ was also similar to a protein encoded by the open reading frame *bpss1385* from *B. pseudomallei* strain K96243 (26% identity). *B. pseudomallei* is the causative agent of melioidosis, an important cause of sepsis in east Asia and northern Australia [14]. Putative Cif homologs were also detected in two *Photorhabdus* species: *P. luminescens*, a symbiotic bacterium for the soil nematode *Heterorhabditis* and a pathogen for a broad range of insects [15] and *P. asymbiotica*, an emerging human pathogen [16]. The proteins encoded by *plu2515* (*P. luminescens*) and *pha4011* (*P. asymbiotica*) share 23 and 26% of identity with Cif_Ec_ respectively. Interestingly, these four bacterial species in which *cif_Ec_*-like genes were found all possess at least one T3SS. Proteins Ypk1971, Bpss1385, Plu2515 and Pha4011 are hereafter referred to as Cif_Yp_, Cif_Bp_, Cif_Pl_ and Cif_Pa_ respectively. Finally, it should also be noted that a truncated putative protein (GOS5485515) obtained from the translation of a DNA fragment isolated from surface water marine samples [17], [18] also shows sequence similarity to Cif_Ec_.

**Table 1:** Cif homologs characterization and sequence conservation with Cif_Ec_.

Proteins	Bacterial origin	To Cif from *E. coli*		GC content (%)		Accession number
		Identity (%)	Similarity (%)	*cif* genes	whole genome	
Cif_Ec_	*Escherichia coli*	-	-	41	50	AAQ07241
Cif_Yp_ (Ypk1971)	*Yersinia pseudotuberculosis*	56	72	43	47	ACA68260
Cif_Bp_ (Bpss1385)	*Burkholderia pseudomallei*	26	45	51	68	CAH38859
Cif_Pl_ (Plu2515)	*Photorhabdus luminescens*	23	42	33	43	CAE14889
Cif_Pa_ (Pha4011)	*Photorhabdus asymbiotica*	26	46	30	42	n/a
GOS5485515	n/a	51	66	n/a	n/a	ECE73989
		(compared to the first 159 residues)				

**Figure 1:**
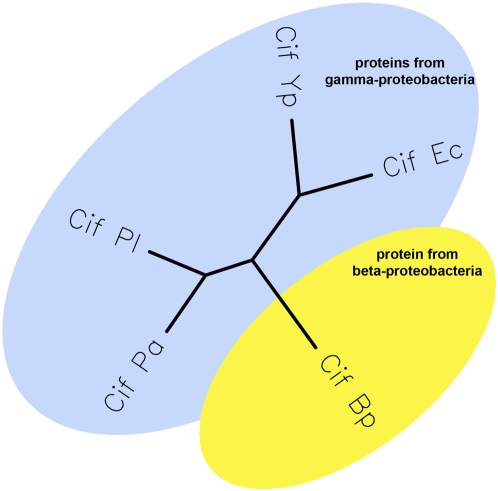
Phylogenetic relationship between Cif_Ec_, Cif_Yp_, Cif_Bp_, Cif_Pl_ and Cif_Pa_. A multiple alignment of the protein sequences (see Fig. 3A) was used to obtain the unrooted tree with Phylip's DrawTree software.

The degree of conservation and the phylogenetic relationship between Cif homologs were analysed by constructing a multiple sequence alignment and a phylogenetic tree using the Neighbour-Joining method (Fig. 1). Cif_Pl_ and Cif_Pa_ clustered together and were separated from a second group consisting of Cif_Ec_ and Cif_Yp_. Cif_Bp_ was the most divergent protein, located to a branch between the two groups. This phylogenetic tree matches the accepted bacterial taxonomy since *B. pseudomallei* belongs to the β-proteobacteria class whereas all others are enterobacteriacae belonging to the γ-proteobacteria class.

### Genes encoding Cif-like proteins are found in highly rearranged DNA regions

**Figure 2:**
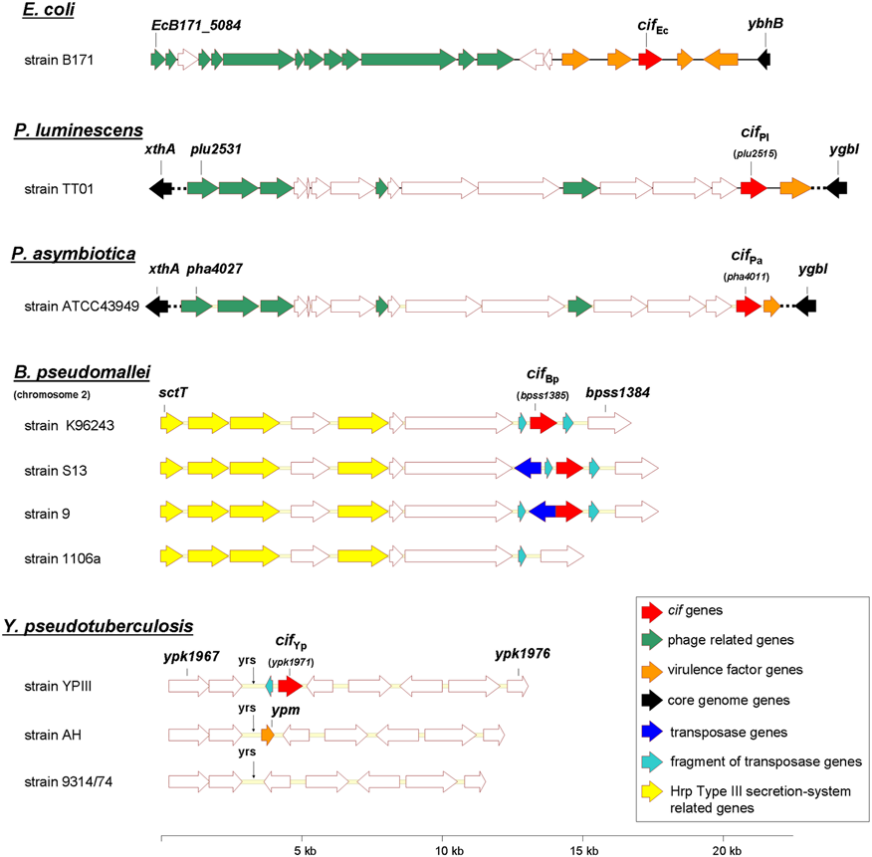
Genetic organization of the *cif*-like genes loci from *E. coli* strain B171, *P. luminescens* strain TT01, *P. asymbiotica* strain ATCC43949, *B. pseudomallei* strains K96243, S13, 9 and 1106a and *Y. pseudotuberculosis* strains YPIII, AH and 9314/74. Open reading frames (ORFs) are represented by horizontal arrows and designation of first and last ORF from each schematic are indicated. Vertical arrows indicate position of the *yr*s sequence (Yersinia recombination site).

In *E. coli*, the *cif* gene is located on an inducible lambdoid prophage spread widely amongst EPEC and EHEC strains (Fig. 2) [11]. In *Photorhabdus* strains, *cif_Pl_* and *cif_Pa_* are located downstream of a region displaying a high degree of similarity to a prophage described in *Serratia entomophila* (Fig. 2) [19]. This prophage is integrated 5 to 6 times in the genome of both *Photorhabdus* species [20] and encodes genes for several putative virulence factors, notably a putative T3SS effector protein homologous to YopT from *Yersiniae*. This phage has no homology with the lambdoid prophage found in *E. coli* isolates but displays some similarity to bacteriocins and R-type pyocins [21]. In *B. pseudomallei* strain K96243 (Fig. 2), *cif_Bp_* is located between two vestigial transposase genes on chromosome II near the *hrp* cluster, which codes for one of the three T3SS present in *B. pseudomallei* [22]. Comparison of sequenced genomes from different *B. pseudomallei* strains reveals that the organization of this locus is highly variable. *B. pseudomallei* strains S13 and 9 contain additional genes, encoding putative transposases, which are inserted near *cif_Bp_* (Fig. 2). In *B. pseudomallei* strain 1106a, this region is deleted and *cif_Bp_* is absent. These data suggest that DNA transposition events could have lead to the heterogeneous distribution of the *cif_Ec_*-like gene in *B. pseudomallei* strains. Among the sequenced strains of *Y. pseudotuberculosis*, only the strain YPIII possesses a gene with similarity to *cif_Ec_*. Comparison of the genetic environment between YPIII and other *Y. pseudotuberculosis* strains revealed that *cif_Yp_* is positioned within a chromosomal locus previously described as the insertion site of *ypm*, a gene coding for a superantigenic toxin in strain AH [23]. Both *ypm* and *cif_Yp_* are located downstream of a 26-bp sequence called *yrs* which is homologous to *dif*, a site-specific recombination target used by filamentous bacteriophages for host chromosome integration. Deletions in the *yrs* locus occur at a higher frequency compared to others regions within the chromosome [23]. Genetic instability at this locus could explain the heterogeneous distribution of both *cif_Yp_* and *ypm* genes in the *Y. pseudotuberculosis* population.

In conclusion, each *cif_Ec_*-like gene is associated either with mobile genetic elements, such as phages, or is located in region of the genome prone to rearrangements, suggesting acquisition of *cif* by horizontal gene transfer in all these bacterial species. This is in agreement with the observation that each *cif_Ec_*-like gene has a different GC content compared to that of their cognate host genome (Table 1).

### Cif proteins share common conserved motifs, including a catalytic triad

**Figure 3:**
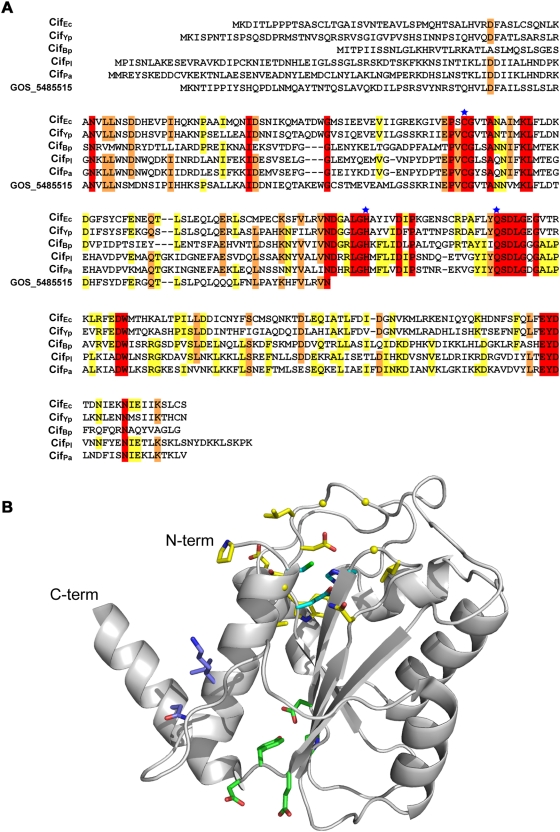
The three residues of the Cif_Ec_ catalytic triad are conserved among members of the Cif protein family. (A) ClustalW alignment between Cif_Ec_, Cif_Yp_, Cif_Bp_, Cif_Pl_, Cif_Pa_ and GOS_5485515. Fully conserved residues are indicated by a red background and amino acids conserved more than 60 or 80% are indicated by a yellow or an orange background respectively. The cysteine, histidine and glutamine residues that form the catalytic triad of Cif_Ec_ are indicated with blue stars. (B) Position of the fully conserved residues in the three dimensional structure of Cif_Ec_. Side chain carbon atoms of residues comprising the catalytic triad are coloured cyan. The remaining fully conserved residues cluster in three regions, as described in the text. Residues coloured yellow, including glycine positions indicated by spheres, are P107, G110, A113, N159, L163-G164, S186-G189, G191, D200-W201; in green are D170, D172, E264-D266; in purple are K118-L119 and N273.

Database searches with the sequence of Cif_Ec_ or the Cif homologs (including the truncated protein from the marine metagenome) reveals no significant matches to well-characterized proteins or motifs. However, alignment of Cif_Ec_ and the homolog sequences reveals several well conserved positions or regions, most of which are located to the C-terminal two thirds of the proteins (Fig. 3A). The lack of sequence conservation at the far N-terminus (top panel in Fig. 3A) is consistent with the putative function of these regions as a translocation signal for the T3SS, which may have different requirements in the different parent organisms. It is now well established that regions responsible for secretion/translocation and chaperone binding in T3SS effectors are located to the N-terminus, but often share no sequence similarity, even for effector proteins translocated by the same T3SS [24].

Sequence alignments of the Cif protein family identified a conserved cysteine residue. Conservation of cysteine residues often implies biological significance. The recent crystal structure of Cif_Ec_ revealed that C109 forms part of a catalytic triad. Further, the other two residues that form this structural motif (H165 and Q185) are also fully conserved in all Cif homologs (Fig 3A). In addition to the catalytic triad, several other residues are also retained. The position of these residues, when mapped onto the structure of Cif_Ec_ (possible for all but three of the conserved residues which are not present in the construct crystallised) reveals they cluster in three regions (Fig. 3B). The first of these clusters surrounds the active site and these residues are likely essential for retaining the catalytic triad in a suitable conformation to enable catalysis, or are directly involved in substrate binding (residues with carbon atoms coloured yellow in Fig. 3B). The second cluster (residues with carbon atoms coloured green in Fig. 3B) is somewhat distant from the active site and it seems likely that this region is important for maintaining structural integrity, with a potentially important hydrogen bond identified between the O^δ1^ atom of Asp170 (located at the end of β-strand 2) and the OH atom of Tyr265 (located at the end of β-strand 4). The importance of the third cluster (of three residues) is less apparent (residues with carbon atoms coloured purple in Fig. 3B). This region may be involved in binding substrate molecules, or it may interact with the N-terminal region of Cif_Ec_ not present in the crystallised protein. In conclusion, *in silico* analyses of Cif homologs are consistent with a conserved function for these proteins, akin to Cif_Ec_.

### Cif_Bp_ is injected by the EPEC T3SS and induces cell cycle arrest and stress fibre formation in HeLa cells

**Figure 4:**
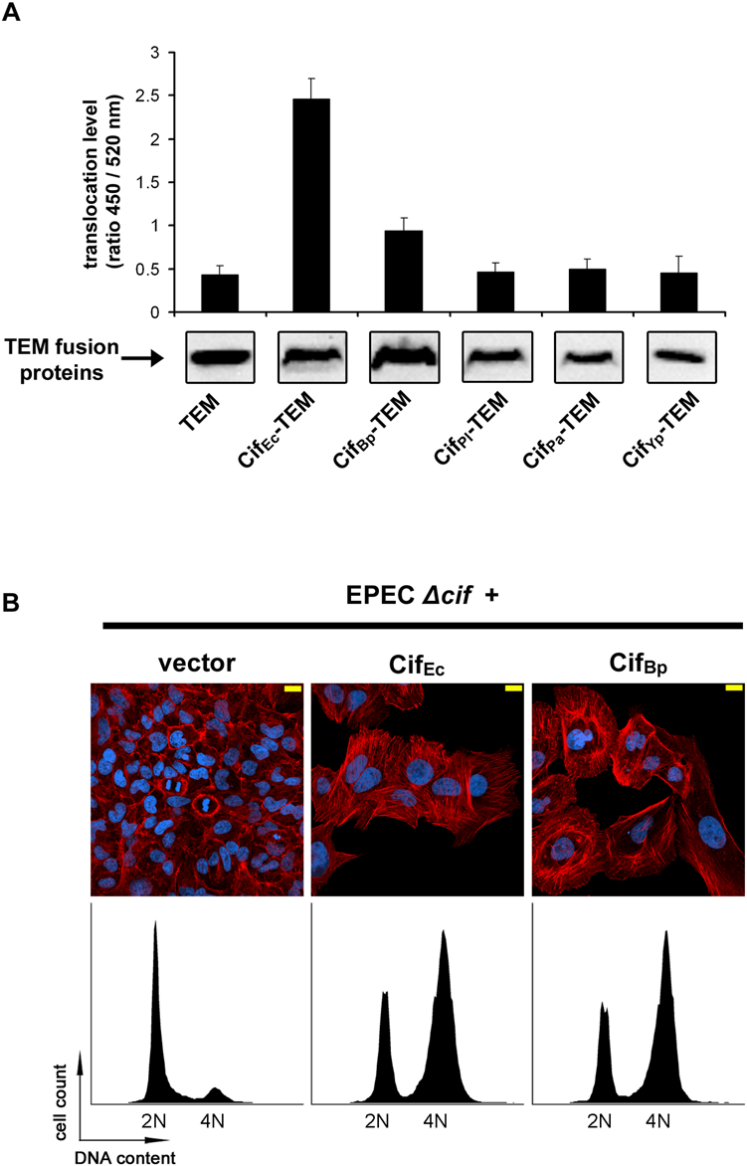
Cif_Bp_ is injected by the EPEC T3SS and induces cell cycle arrest and stress fibre formation in HeLa cells. (A) Translocation of Cif_Ec_-TEM, Cif_Bp_-TEM, Cif_Pl_-TEM, Cif_Pa_-TEM and Cif_Yp_-TEM fusions by the T3SS of EPEC strain E22. Hela cells were loaded with CCF2/AM substrate and were infected for 2 and a half h with E22*Δcif* hosting plasmids expressing TEM alone or the different Cif-TEM fusions. Upper panel: intracellular β-lactamase activity detected by measuring cleavage of the CCF2/AM, as described in Material and Methods. This ratio represents the relative translocation efficiency [9]. Experiments were performed in triplicate and error bars represent standard errors of the mean. Lower panel: synthesis of TEM fusions proteins were quantified in bacteria just before the translocation assays by western blot with anti-TEM antibodies. (B) G_1_/S synchronized HeLa cells were exposed for 90 min to E22*Δcif* hosting either empty vector or the plasmids expressing Cif_Ec_ or Cif_Bp_, washed and incubated with antibiotic for 20 or 72 h. Upper panels: F-actin was labelled with phalloidin-rhodamine (red) and DNA with DAPI (blue) 72 h post-infection. Bars represent 20 µm. Lower panels: cell cycle distribution was analysed by flow cytometry 20 h post-infection. 2N and 4N populations are indicated.

An EPEC strain deleted for its chromosomal *cif_Ec_* gene (E22Δ*cif*) has previously been described [3]. To test whether the Cif-like proteins are functional homologs of Cif_Ec_, the E22Δ*cif* strain was complemented with a plasmid encoding each of the *cif*_Ec_-like genes, and these bacteria were used to infect cultured HeLa cells. Since the whole amino acid sequence of the putative protein derived from the marine metagenome is not available, this truncated protein was not included in these assays. Before phenotypic characterization of cells infected with EPEC producing the Cif homologs, the translocation efficiency of the proteins by the EPEC T3SS was monitored using the TEM/CCF2 assay [9]. As expected, the Cif_Ec_-TEM fusion protein was properly translocated, as demonstrated by detection of intracellular β-lactamase activity (Fig. 4A). TEM activity was also detected in cells infected with E22*Δcif* producing Cif_Bp_-TEM, but levels of β-lactamase activity for Cif_Pl_-TEM, Cif_Pa_-TEM and Cif_Yp_-TEM were similar to the basal level detected with the negative control (TEM alone, Fig. 4A). Since TEM fusion proteins were produced to similar levels in the bacteria (Fig. 4A), absence of intracellular TEM activity likely results from inefficient recognition and/or injection of Cif_Pl_-TEM, Cif_Pa_-TEM and Cif_Yp_-TEM by the T3SS of EPEC. The lower translocation level of Cif_Bp_-TEM compared to Cif_Ec_-TEM probably also reflects a poor recognition of the secretion/translocation signal (STS) of Cif_Bp_ by the T3SS from EPEC. Indeed, when this fusion protein was expressed in an *escN* mutant (T3SS ATPase defective mutant), β-lactamase activity was no longer detected in infected cells, confirming that translocation of Cif_Bp_-TEM by E22 strain is T3SS-dependent (data not shown).

Since Cif_Bp_ can be injected by the T3SS of E22, the capacity of the protein to induce cytopathic phenotypes on HeLa cells was analysed using the infection model. In contrast to cells infected with E22*Δcif* carrying an empty vector, cells infected with E22*Δcif* producing Cif_Bp_ developed cell distension and actin stress fibres indistinguishable from those induced by a Cif_Ec_-expressing strain (Fig. 4B). Cif_Bp_ also blocked cell cycle progression, as demonstrated by the accumulation of G_2_ arrested cells containing 4N DNA content (Fig. 4B). These phenotypes were not induced when Cif_Bp_ was expressed in an *escN* mutant (data not shown). These data clearly demonstrate that Cif_Bp_ is a functional homolog of Cif_Ec_.

### Cif_Pl_, Cif_Pa_ and Cif_Yp_ are functional homologs of Cif_Ec_

**Figure 5:**
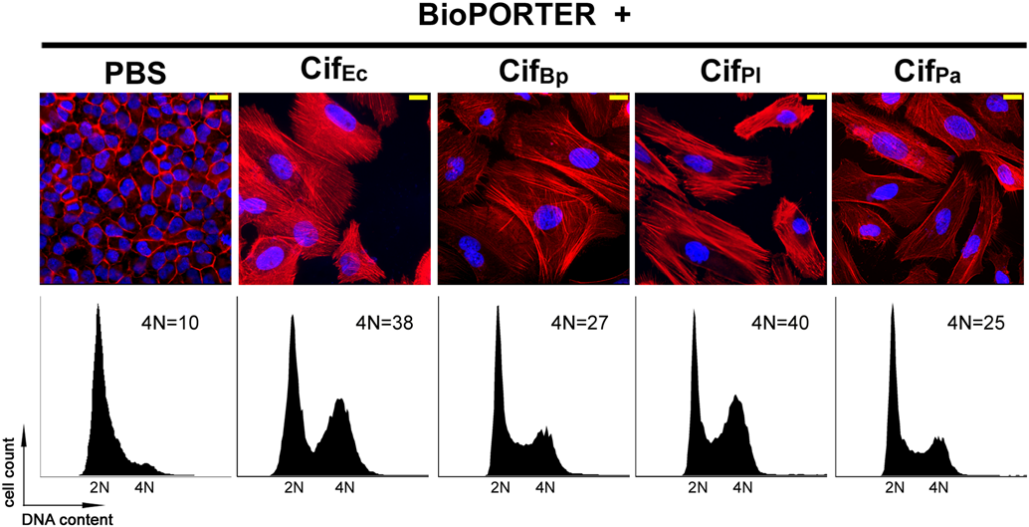
Lipofection of purified Cif_Bp_, Cif_Pl_ and Cif_Pa_ proteins into HeLa cells induce cell cycle arrest and stress fibres formation akin to Cif_Ec_. G_1_/S synchronized HeLa cells were treated with purified proteins or PBS in combination with a lipidic delivery agent (BioPORTER). Upper panels: F-actin was stained with phalloidin-rhodamine (red) and DNA with DAPI (blue) 72 h post-treatment. Bars represent 20 µm. Lower panels: cell cycle distribution was analysed by flow cytometry 20 h post-treatment. Percentages of cells with 4N DNA content are indicated.

As the EPEC T3SS was not able to translocate Cif_Pl_, Cif_Pa_ and Cif_Yp_ into infected cells, the cytopathic activity of these Cif_Ec_-like proteins was investigated using purified recombinant samples combined with a lipid mediated delivery system (BioPORTER) as previously described [5]. The effects of Cif_Bp_ delivered with this system were also investigated. Cif_Bp_, Cif_Pl_ and Cif_Pa_ were all readily overexpressed and purified in a soluble form (see Materials and Methods). However, despite many efforts, it was not possible to obtain a purified soluble form of Cif_Yp_ at levels necessary for activity assays using the BioPORTER delivery system. As previously reported [5], treatment of HeLa cells with BioPORTER mixed with purified Cif_Ec_ leads to cell enlargement and formation of actin stress fibres (Fig. 5), identical to the phenotype observed with the infection model. However, as protein delivery with BioPORTER is not as efficient as bacterial infection [5], only ∼50% of the treated cells exhibit morphological alterations (not shown). Studies of cell cycle patterns were therefore realized using G_1_/S synchronized cells to improve visualization of G2 arrest. In contrast to cells incubated with the lipid delivery agent mixed with PBS alone, cells treated with BioPORTER+Cif_Ec_ accumulated in G2 phase (38% of Cif_Ec_-treated cells contained 4N DNA-content against 10% for PBS-treated cells). Lipofection of purified Cif_Bp_ into HeLa cells led to actin stress fibres and cell accumulation in G_2_ phase (27%) (Fig. 5), confirming the functionality of Cif_Bp_ observed with the infection assays. Introduction of purified Cif_Pl_ or Cif_Pa_ into HeLa cells with BioPORTER also led to cell enlargement, cytoskeleton alteration and accumulation of cells with 4N DNA content (40 and 25% for Cif_Pl_ and Cif_Pa_ respectively versus 10% for PBS treated cells, see Fig. 5). Therefore, Cif_Pl_ and Cif_Pa_ are also functional homologs of Cif_Ec_. As these phenotypes are observed with purified proteins, the results demonstrate that the proteins alone are sufficient to induce the Cif-associated cytopathic effects.

**Figure 6:**
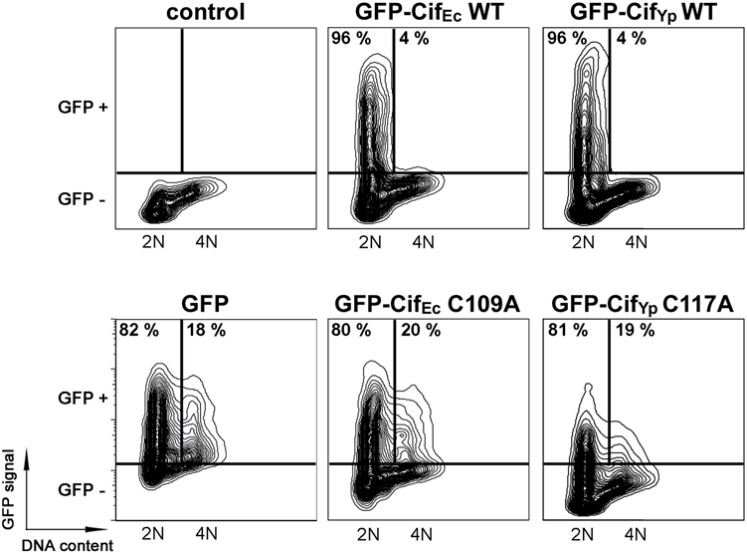
Transfection of Cif_Yp_ induces cell cycle arrest in HeLa cells. HeLa cells were transfected with plasmid expressing GFP, GFP-Cif_Ec_ or GFP-Cif_Yp_ (wild-type (WT) or Cys variants (C/A)) fusion proteins. GFP expression and DNA content were analysed by flow cytometry 48 h post-transfection. Data are represented on two dimensional contour plot graphics with DNA content on the X-axis and GFP signal on the Y-axis. Gates corresponding to the GFP negative and GFP positive populations are indicated. Among the GFP positive populations, percentages of cells with 2N or 4N DNA content are indicated within the corresponding quadrants.

As it was not possible to introduce Cif_Yp_ into cells using either the infection or BioPORTER treatments, the function of this protein was analysed directly by expressing *cif*_Yp_ in HeLa cells. Cif_Yp_ and Cif_Ec_, used as a positive control, were expressed as a translational fusion with the fluorescent reporter protein GFP, allowing quantification of GFP-Cif expression in transfected cells. GFP alone was also transfected as a negative control. Among the GFP positive population, 96% of cells expressing GFP-Cif_Ec_ had a 2N DNA content whereas the 2N population of cells expressing GFP alone was only 82% (Fig. 6). Consistent with previous studies demonstrating that Cif could also induce G_1_/S arrest [6], this result demonstrates that the cell cycle of transfected cells expressing GFP-Cif_Ec_ was blocked in G_1_ (2N DNA content). As expected, the cell cycle arrest was not observed when the critical cysteine residue from the catalytic triad of Cif_Ec_ was substituted (Fig. 6). Expression of GFP-Cif_Yp_ in HeLa cells also led to accumulation of GFP-positive cells with 2N DNA content (96% against 82% for cells expressing GFP alone), demonstrating that Cif_Yp_ induced a cell cycle arrest in G_1_ phase similarly to Cif_Ec_ (Fig. 6). This result indicates that Cif from *Y. pseudotuberculosis* is a functional homolog of Cif_Ec_.

### The conserved catalytic triad is critical for the activity of Cif homologs

**Figure 7:**
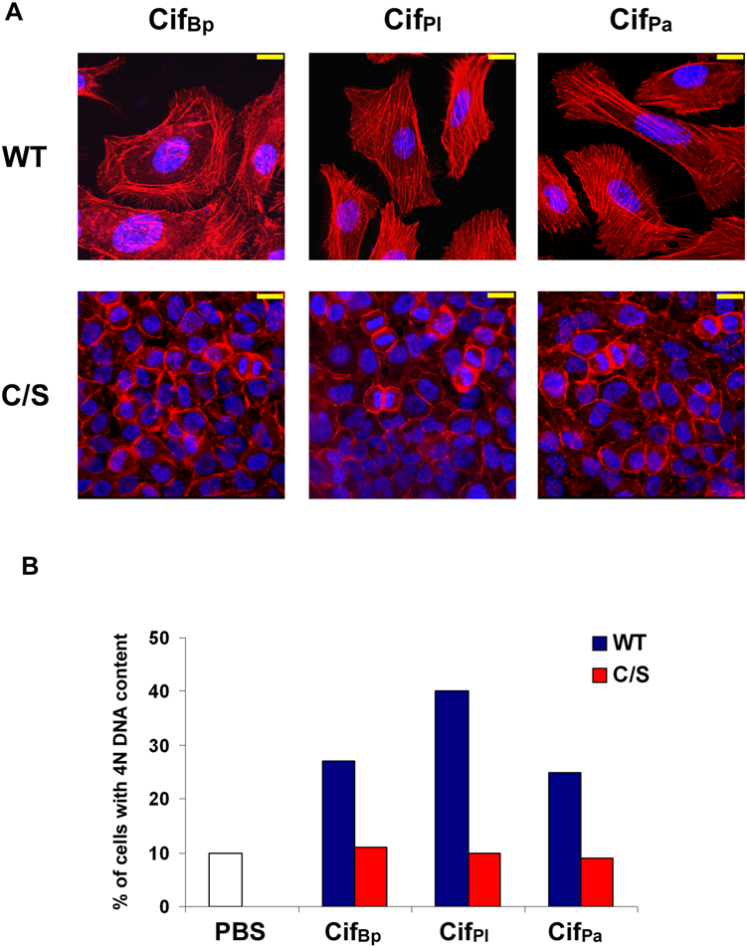
Cysteine residues from predicted catalytic triads are essential for Cif homologs activity. (A) HeLa cells were treated with purified Cif homologs proteins (wild-type (WT) or cysteine variants (C/S)) in combination with a lipidic delivery agent (BioPORTER). F-actin was stained with phalloidin-rhodamine (red) and DNA with DAPI (blue) 72 h post-treatment. Bars represent 20 µm. (B) G_1_/S synchronized HeLa cells were treated with PBS or purified Cif proteins (as indicated), in combination with BioPORTER. The percentages of the populations containing 4N DNA content were determined by flow cytometry 20 h post-treatment.

Most of the conserved residues in Cif proteins are clustered in discrete regions (Fig. 3). The cysteine, histidine and glutamine residues forming the catalytic triad in Cif_Ec_ were shown to be critical for activity [10]. To determine whether an equivalent functional catalytic site exists in the Cif homologs, the conserved cysteines in Cif_Bp_, Cif_Pl_ and Cif_Pa_ (C90, C128 and C123 respectively) were substituted with a serine residue, and the corresponding proteins were purified prior to delivery into HeLa cells using the BioPORTER system. In contrast to the wild-type proteins, the cysteine variants did not induce cell enlargement and stress fibre formation (Fig. 7A). Further, analysis of DNA content revealed that accumulation of G2-arrested cells did not occur when cells were treated with the cysteine variants (Fig. 7B). Expression of the cysteine variant from Cif_Yp_ by transfection in HeLa cells also revealed that the cell cycle was not arrested in contrast to cells producing the wild-type protein (Fig. 6). These results demonstrate that the conserved cysteine residue is critical for Cif activity. Also, as the histidine and glutamine residues that complete the triad are also conserved in the sequences of the Cif homologs, this suggests that catalytic triads also exist in Cif_Bp_, Cif_Pl_, Cif_Pa_ and Cif_Yp_.

### Cif homologs induce p21 and p27 accumulation in cells

**Figure 8:**
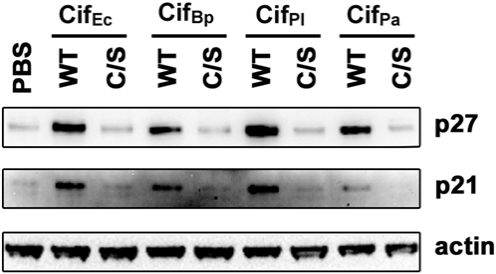
Cif homologs induce p21 and p27 accumulation in cells. HeLa cells were treated with PBS or purified Cif proteins (wild-type (WT) or cysteine variants (C/S) as indicated), in combination with BioPORTER. Cell extracts were probed with anti-p21, anti-p27 and anti-actin antibodies 24 h post-treatment.

It has recently been shown that the cytopathic activity of Cif_Ec_ is correlated to the accumulation of CKIs p21 and p27, two important regulators of cell cycle progression [6]. Since all Cif homologs appear to share the same catalytic triad and induce identical cytopathic phenotypes in HeLa cells, we wonder if they could hijack the same signaling pathways, despite the fact that two of these proteins are produced by bacteria colonizing insects and nematodes. Western-blot analysis of HeLa cells treated with purified Cif homologs indicated that levels of p21 and p27 increase in the presence of wild-type Cif_Bp_, Cif_Pl_ and Cif_Pa_ (Fig. 8). An intact catalytic triad is integral to this accumulation as CKIs levels were not affected when cells were treated with the cysteine variants (Fig. 8). This accumulation of p21 and p27 suggests that the molecular mechanisms involved in Cif cytotoxicity on HeLa cells are identical for Cif_Ec_ and the Cif homologs.

## Discussion

Cif_Ec_ proteins belong to a family of cyclomodulins that inhibit host cell proliferation by inducing G_1_/S and G_2_/M phase transition blocks [3], [6]. In this study, functional homologs of Cif from pathogenic *E. coli* have been identified in *Y. pseudotuberculosis*, *B. pseudomallei*, *P. luminescens* and *P. asymbiotica*. These homologs possess the same capacity as Cif_Ec_ to induce cell cycle arrest, actin stress fibre formation and p21 and p27 CKIs accumulation when introduced into HeLa cells, suggesting they target the same substrates. Each of the Cif homologs possesses a predicted catalytic triad as identified in the crystal structure of Cif_Ec_. This triad is involved in the cytotoxic activity of each Cif homolog as substitution of the conserved cysteine residue in any of the proteins leads to inactivation.

In pathogenic *E. coli*, *cif* is located on an inducible lambdoïd prophage that has spread widely within the natural population of *E. coli* [11]. Analysis of the genetic locus containing *cif* in other bacteria reveals that *cif* genes from *Photorhabdus* species are also located downstream of a prophage, while *cif* from *B. pseudomallei* and *Y. pseudotuberculosis* are inserted in highly rearranged DNA regions leading to heterogeneous distribution within bacterial populations. In addition, GC content of *cif* genes shows substantial deviation from the general pattern within their respective genome. In light of these data, *cif* genes are proposed to have been acquired by horizontal gene transfer and could be defined as xenologs according to the nomenclature proposed by Koonin *et al.* [25]. The phylogenetic relationship between the different xenologs is in agreement with the bacterial taxonomy since Cif from *B. pseudomallei*, the only β-proteobacteria, is the most divergent protein. This indicates that protein sequence variation is, to some extent, a consequence of speciation events and suggests that *cif* genes were probably acquired early during bacterial evolution. All Cif-producing bacteria encode at least one T3SS that could inject the effector into host cells during infection. It is interesting to speculate that tight association between horizontally acquired effectors and the T3SS machinery in bacteria is a consequence of selective pressure since advantages conferred by effector acquisition will occur only if the recipient organism produces the secretion/translocation machinery. Such an association has already been described in *E. coli* and *Salmonella enterica* serovar Typhimurium where phage-encoded T3SS effectors were associated with T3SS producing isolates [11], [26].

Bacteria habouring the *cif* gene spend part of their life cycle in association with eukaryotic organisms. While *E. coli*, *B. pseudomallei*, *Y. pseudotuberculosis* and *P. asymbiotica* are mammalians pathogens [13], [14], [16], [27], both *Photorhabdus* species are pathogenic for insects and symbiotic to nematodes [15], [28], [29]. Like the Cif proteins, other families of T3SS effectors are produced by bacterial pathogens that target distinct hosts. For example, a number of proteins belonging to the YopT cysteine protease family have been described in mammalian, insect and plant pathogens [30]. Although the overall sequence identity at the amino acid level is not extensive, every member of the YopT family shows several invariant residues including a cysteine, a histidine and an aspartate that form a putative catalytic triad. Representatives from the YopT-like family interfere with diverse host immune responses and display protease activity dependent on an intact catalytic triad. YopT, the archetypal member of this family, is the most potent inhibitor of phagocytosis produced by *Yersinia* [31] and cleaves prenylated GTPases of the Rho family in host cells [32]. Similar to YopT, LopT from *P. luminescens* is able to release RhoA from human and insect cell membranes [33]. AvrPphB is an avirulence protein of the YopT-like family from the plant pathogen *Pseudomonas syringae* that triggers a disease-resistance response in a number of host plants, including Arabidopsis [30]. Searches of the Protein Data Bank with the structure of Cif_Ec_ reveal close structural homology to AvrPphB. Although the residues that form the catalytic triad in each protein are different (C/H/D for AvrPphB and C/H/Q for Cif), the overall folds and residues comprising the catalytic triads superimpose well [10].

YopJ-like proteins form a second family of T3SS effectors produced by different animal and plant pathogens that also possess conserved residues forming a predicted catalytic triad, which is required for protease activity [34]. YopJ, the archetypal member of this family, is an essential virulence factor produced by *Yersinia* which blocks MAPK and NFκB pathways resulting in inhibition of host immune responses [35], [36]. In contrast to members of the Cif protein family, that induce similar phenotypes in HeLa cells, proteins belonging to the YopT or the YopJ family appear to generate different responses in eukaryotic cells. For example, AvrA, a *Salmonella* YopJ-like T3SS effector (56% identity with YopJ), does not induce the same host responses observed for YopJ [37]. Further studies are required to determine whether the conserved cytopathic effects induced by Cif proteins in HeLa cells, notably cell cycle arrest, also occur in their respective host cells (gut enterocytes for intestinal pathogens, insect cells for *Photorhabdus* species, etc).

Interestingly, the plant symbiont *Rhizobium* sp. strain NGR234 produces NopJ, a YopJ-like protein and NopT, a T3SS effector belonging to YopT-like family [38]–[40]. Both cysteine proteases were shown to be involved in the host-specific nodulation response of legumes [38], [40]. Symbiotic bacteria deploy somewhat similar strategies for colonizing host cells as those used by mammalian pathogens. The T3SS is, for example, required for host cell invasion by a variety of symbiotic bacteria [41]–[43]. As the Cif-producing *Photorhabdus* species are not only insect pathogens but also nematode symbionts, it is tempting to speculate that Cif may also contribute to the symbiotic process. Further, CdtB, the active monomer of the cyclomodulin CDT, is expressed in *Hamiltonella defensa*, a symbiont of pea aphids [44]. It maybe that symbionts use cyclomodulins like CDT and Cif to modulate, rather than globally deregulate, host signaling pathways resulting in initiation of symbiosis. Future studies will rely on further molecular (*in vitro*) analysis and *in vivo* models to achieve a full understanding of the roles of Cif in microbial pathogenesis, commensalism and symbiosis.

## Materials and Methods

### Cell line, bacterial strains and plasmids

HeLa cells (ATCC CCL-2) were cultured in Dulbecco's modified Eagle medium (DMEM; Invitrogen) supplemented with 10% foetal calf serum (FCS; Eurobio) and 80 µg ml^−1^ gentamicin at 37°C in a 5% CO_2_ atmosphere. For synchronization in G_1_/S phase, HeLa cells were treated with 2 mM thymidine (Sigma) for 18 h, washed 3 times with Hank's balanced salt solution (HBSS; Invitrogen), incubated in normal medium for 9 h and treated again with 2 mM thymidine for 16 h. Bacterial strains and plasmids used in this study are listed in Table 2. Bacteria were cultured in Luria-Bertani (LB) broth or in interaction medium (DMEM with 25 mM Hepes and 5% FCS). Antibiotics were used at the following final concentrations: chloramphenicol 20 µg ml^−1^ and kanamycin 25 µg ml^−1^.

**Table 2:** *E. coli* strains and plasmids used in this study.

Strains and plasmids	Genotype or description	Reference
**strains**		
E22Δ*cif*	Rabbit EPEC Δ*cif::frt*	[3]
DH5α		Invitrogen
BL21-CodonPlus (DE3)		Stratagene
**Plasmids**		
pBRSK	Cloning vector derived from pBR328	[45]
pEL1	pBRSK expressing Cif_Bp_	This study
pEL2	pBRSK expressing Cif_Pl_	This study
pEL3	pBRSK expressing Cif_Ec_	[5]
pEL4	pBRSK expressing Cif_Pa_	This study
pEL5	pBRSK expressing Cif_Yp_	This study
pBBR1MCS-2	Low-copy cloning vector	[46]
pKTEM	TEM-1 fusion cloning vector derived from pBBR1MCS-2	This study
pGJ626	pKTEM expressing Cif_Ec_-TEM fusion	This study
pGJ719	pKTEM expressing Cif_Bp_-TEM fusion protein	This study
pGJ720	pKTEM expressing Cif_Pl_-TEM fusion protein	This study
pGJ721	pKTEM expressing Cif_Pa_-TEM fusion protein	This study
pGJ803	pKTEM expressing Cif_Yp_-TEM fusion protein	This study
pET28a	Protein expression vector	Novagen
pMB1	pET28a expressing 6xHis-Cif_Bp_ fusion protein	This study
pMB2	pET28a expressing 6xHis-Cif_Bp_ C90S fusion protein	This study
pCC1	pET28a expressing 6xHis-Cif_Pl_ fusion protein	This study
pCC2	pET28a expressing 6xHis-Cif_Pl_ C128S fusion protein	This study
pCC3	pET28a expressing 6xHis-Cif_Pa_ fusion protein	This study
pCC4	pET28a expressing 6xHis-Cif_Pa_ C123S fusion protein	This study
pTagGFP-C	Cloning vector for GFP translational fusion expression	Evrogen
pRN1	pTagGFP-C expressing GFP-Cif_Ec_ WT	This study
pRN2	pTagGFP-C expressing GFP-Cif_Ec_ C109A	This study
pGJ808	pTagGFP-C expressing GFP-Cif_Yp_ WT	This study
pGJ809	pTagGFP-C expressing GFP-Cif_Yp_ C117A	This study

### Construction of plasmids expressing Cif and Cif-TEM proteins

To construct plasmids suitable for expressing Cif homologs from *B. pseudomallei*, *Y. pseudotuberculosis*, *P. luminescens* and *P. asymbiotica* in EPEC, *cif*_Bp_, *cif*_Yp_, *cif*_Pl_ and *cif*_Pa_ genes were amplified from respective genomic DNA with primers adding a *Xba*I restriction site at the start codon and a *Bam*HI (or *Xho*I for *cif*_Bp_) restriction site after the stop codon. PCR products were digested and ligated into the corresponding sites of the pBRSK vector [45]. The resulting plasmids pEL1, pEL2, pEL4 and pEL5 contain, respectively, *cif*_Bp_, *cif*_Pl_, *cif*_Pa_ and *cif*_Yp_ genes under the control of a P*lac* promoter.

To create the pKTEM vector necessary to construct TEM fusions, the multiple cloning site and *blaM* gene (encoding the β-lactamase TEM-1) were amplified by PCR from pCX340 ([9]) with primers containing *Xho*I and *Xba*I restriction sites. The PCR fragment was digested and cloned into the corresponding sites of pBBR1MCS-2 ([46]). Plasmids encoding translational fusion between the different Cif proteins and the β-lactamase TEM-1 were obtained by cloning *cif* genes into the pKTEM vector. Briefly, *cif*_Bp_, *cif*_Pl_, *cif*_Ec_, *cif*_Pa_ and *cif*_Yp_ genes were amplified from pEL1, pEL2, pEL3, pEL4 and pEL5 respectively using primers with *Nde*I-*Eco*RI restriction sites (or *Xho*I-*Hind*III for pEL4), digested and cloned into the corresponding sites of pKTEM. The resulting plasmids pGJ719, pGJ720, pGJ626, pGJ721 and pGJ803 encode Cif_Bp_-TEM, Cif_Pl_-TEM, Cif_Ec_-TEM, Cif_Pa_-TEM and Cif_Yp_-TEM fusion proteins respectively. All the constructs were verified by DNA sequencing (Cogenics, France).

### Purification of Cif_Bp_, Cif_Pl_, Cif_Pa_ and Cif_Ec_ proteins

For production of recombinant protein, the genes encoding *cif*_Bp_, *cif*_Pl_, *cif*_Pa_ and *cif*_Yp_ were cloned into the pET28a vector (Novagen). The resulting constructs encoded proteins with an N-terminal 6xHis tag. Plasmids were named pMB1, pCC1, pCC3 and pGJ803 respectively. The plasmid for expression of 6xHis-Cif_Ec_ has been described elsewhere [5]. Mutations of the conserved cysteine residues were obtained by inverse PCR using pET28 based constructs as a template and oligonucleotides containing specific base changes. All the constructs were verified by DNA sequencing (Cogenics, France). After transformation into the *E. coli* BL21-CodonPlus® (DE3)-RIPL strain (Stratagene), bacteria were grown in LB to an OD_600 nm_ of ∼0.6 then induced with 0.5 mM IPTG for 3 h at 37°C. Purification of native proteins was achieved by Ni-NTA chromatography as recommended by the manufacturer (Qiagen) and, if necessary, gel filtration. Samples were then dialysed against PBS, aliquoted and stored at −80°C.

### Construction of plasmids expressing GFP-Cif fusion proteins and transfection assays

Plasmids encoding translational fusions between the fluorescent reporter protein GFP and Cif_Ec_ or Cif_Yp_ were obtained by cloning *cif* genes (encoding the wild-type or the cysteine variant forms) into the pTagGFP-C vector (Evrogen). The resulting plasmids were verified by DNA sequencing (Cogenics, France). Transfections were performed in 6-well plates with FuGENE (Roche) according to the manufacturer's instructions. Two days after transfection, HeLa cells were exposed to trypsin, washed with ice-cold PBS, fixed for 3 h at 4°C in PBS with 1% formaldehyde, permeabilized overnight at 4°C in PBS ethanol 70% and stained with propidium iodide for 30 min at 37°C. Cells were analysed using a FACScalibur flow cytometer (Becton Dickinson) and data from at least 20 000 cells were analysed using FloJo software v8.5 (Tree Star).

### Infection, translocation and BioPORTER assays

For infection experiments, bacterial strains were cultured overnight in LB broth then diluted 1∶100 in interaction medium for 3 h at 37°C in a 5% CO_2_ atmosphere. HeLa cells were washed with HBSS and infected for the indicated time in interaction medium with a multiplicity of infection (MOI) of 100 bacteria per cell (except as otherwise noted). After the infection, cells were washed with HBSS then cultivated for the indicated times in DMEM medium supplemented with 10% FCS and 200 µg ml^−1^ gentamicin.

Translocation levels of Cif-TEM fusion proteins were determined using CCF2/AM (Invitrogen) as a substrate for intracellular TEM enzyme as described previously [9]. Briefly, HeLa cells seeded in black 96-well plates were loaded for 1 h at 37°C with 1.7 µM CCF2/AM diluted in DMEM with 2 mM probenecid and then infected for 2 and a half h with bacteria expressing TEM fusion proteins. Fluorescence was quantified in a microplate reader (TECAN Infinite M200) with excitation set at 410 nm (9 nm bandwidth) and emission at 450 nm for blue fluorescence and 520 nm for green fluorescence (20 nm bandwidth). Translocation was expressed as the emission ratio at 450/520 nm. To determine the expression level of TEM fusion proteins in bacteria, bacterial cultures with identical OD_600 nm_ were pelleted, resuspended in SDS-PAGE sample buffer, boiled for 5 min and subjected to western blot analysis with anti-TEM-1 antibodies (QED Biosciences).

For BioPORTER assays, 80 µl of purified proteins (250 µg ml^−1^) (or PBS as a negative control) were added to one BioPORTER tube (Genlantis) and resuspended in 420 or 920 µl of DMEM. The samples were added to the cells grown in BD Falcon culture slides or in 6-well plates and incubated for 4 h. BioPORTER mixes were replaced by fresh complete medium and the cells were incubated for 16–72 h.

### Actin stress fibre and cell cycle analyses

For cell morphology and actin cytoskeleton visualization, cells were fixed for 15 min in PBS supplemented with 4% formaldehyde, permeabilized with 0.1% Triton X-100 and stained with rhodamine-phalloidin (Molecular Probes) and DAPI (Sigma). Images were acquired with a DMRB fluorescence microscope equipped with a DFC300FX digital camera (Leica). Cell cycle distribution analyses were performed as previously described [47]. Briefly, cells were grown on 6-well plates, synchronized in G_1_/S phase and infected or treated with BioPORTER. The cells were exposed to trypsin, washed, fixed with ethanol, stained with propidium iodide and analyzed by flow cytometry. Percentages of G_2_ populations were calculated using the Dean-Jett-Fox model from the FlowJo software (Tree Star).

### Western Blot analyses

For Western blot analyses, 6×10^5^ cells were lysed in 80 µl of SDS-PAGE sample buffer, sonicated for 2 s to shear DNA and then boiled for 5 min. Protein samples were resolved on 4–12% NuPage gradient gels (Invitrogen) and blotted on PVDF membranes. Membranes were blocked in TBST (10 mM Tris pH 7.8, 150 mM NaCl, 0.1% Tween20) 5% non-fat dry milk, then probed with primary antibody (0.5 µg ml^−1^) in TBST 5% non-fat dry milk. Primary antibodies were: anti-actin (ICN), anti-p21 and anti-p27 (Santa Cruz Biotechnology). Bound antibodies were visualized with horseradish peroxidase-conjugated secondary antibody. Acquisitions were performed with a Molecular Imager ChemiDoc XRS system (Bio-Rad). Protein levels were quantified with Quantity One Software (Bio-Rad) and normalized with actin level.

### Bioinformatic analyses

The search for proteins sharing similarity with Cif_Ec_ was performed using BLAST on the NCBI server and MaGe system on the Genoscope server for private access to the genome of *P. asymbiotica* (Sanger Institute). Genetic organization of the *cif*-like genes loci were determined using Artemis software from the Sanger Institute and MaGe system. Multiple alignments of Cif sequences were generated with ClustalW and edited using GeneDoc software. Based on this alignment, the unrooted phylogentic tree was obtained using Phylip's Draw software.
